# Optimization Approach for Hydrogen Infrastructure Planning Under Uncertainty

**DOI:** 10.1021/acs.iecr.4c04211

**Published:** 2025-03-26

**Authors:** Margarita E. Efthymiadou, Vassilis M. Charitopoulos, Lazaros G. Papageorgiou

**Affiliations:** The Sargent Centre for Process Systems Engineering, Department of Chemical Engineering, UCL (University College London), Torrington Place, London WC1E 7JE, U.K.

## Abstract

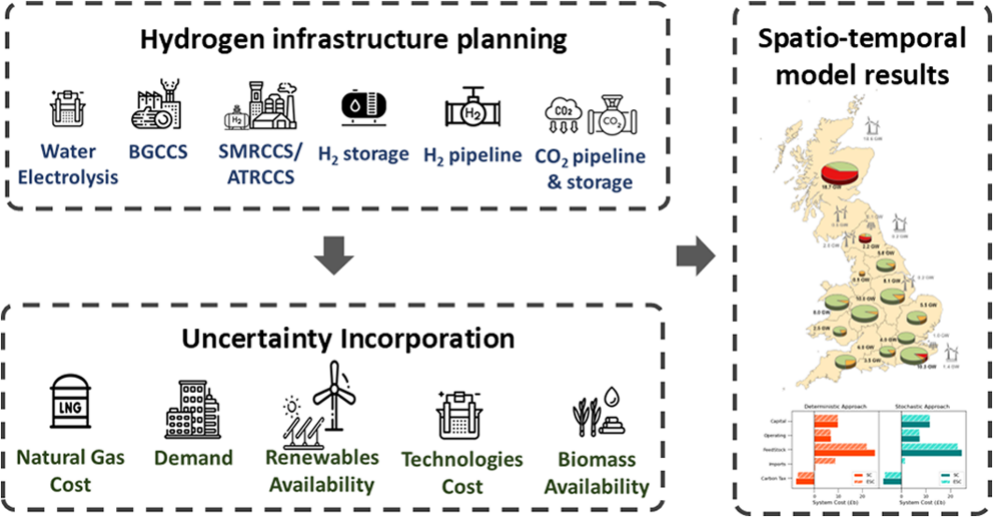

Toward the Net-Zero goal, deciphering trade-offs in strategic decisions for the role of hydrogen is vital for transitioning to low-carbon energy systems. This work proposes a two-stage stochastic optimization framework to provide insights for infrastructure investments in hydrogen production, storage, transmission, and CO_2_ capture and storage. The mixed-integer linear programming (MILP) model aims to minimize total system cost with detailed spatiotemporal resolution to meet hydrogen demand in Great Britain. Uncertainty is considered in hydrogen demand, gas, and technology costs, as well as renewables and biomass availability. To address the resulting combinatorial complexity, scenarios are reduced using forward scenario reduction. Optimization results indicate that a combination of autothermal reforming and biomass gasification with carbon capture and storage (CCS) is the most cost-efficient strategy under uncertainty. A what-if analysis explores the impact of water electrolysis penetration on the production mix. The results demonstrate that considering uncertainties provides a risk-averse strategy for decision-making in low-carbon hydrogen pathways.

## 1 Introduction

Over the last few decades, combustion of fossil fuels has acted as the primary driver behind the increased concentrations of greenhouse gases in the atmosphere. This phenomenon has adverse effects on the planet, including extreme heat, floods, and wildfires, due to global temperature rise by almost 1 °C since the mid-1970s.^1^ In 2023, U.K.’s net greenhouse emissions were estimated to be 384.2 MtCO_2e_, with CO_2_ emissions reduced by 52.7% compared to 1990 levels.^2^

The major emitting sector in the U.K. is transportation, accounting for 29% of all greenhouse gas emissions. Moreover, buildings and industry sectors have a significant impact on the environmental footprint, accounting for 20.2 and 13.7% of total emissions.^2^ However, further reduction is required toward a sustainable and economically efficient pathway to Net-Zero. The U.K. was the first major economy globally that legislated to reach Net-Zero carbon emissions by 2050. A Net-Zero strategy was published setting out clear policies and proposals for a decarbonized economy.^3^ In this context, the U.K. government has set a goal of 75% emissions decrease from public sector buildings by 2037 compared to a 2017 baseline.^4^

Thus, there is an urgency to explore low-carbon alternative pathways, such as hydrogen, ammonia, and methane, to replace carbon-based fuels. However, the complexity of energy systems constitute a great challenge toward the Net-Zero transition.^5^ To address this issue, many works have focused on the introduction of alternative fuels in future energy systems investigating cost-optimal investments while reducing the environmental impact.^6−10^

Hydrogen has emerged as a key vector in decarbonizing energy systems either with its direct use as an alternative to natural gas or as an energy carrier for renewable energy generation. Recognizing the importance of a hydrogen-led economy, the U.K. government has set a target of 5 GW of low-carbon hydrogen production capacity by 2050.^11^ The development of new hydrogen infrastructure networks is inevitable to achieve the aforementioned goal. Consequently, novel modeling tools for the hydrogen supply chain are required to provide useful insights regarding strategical investment decisions.

In the last decades, a considerable body of literature has emerged addressing hydrogen supply chain infrastructure design using optimization-based approaches. More specifically, mixed-integer linear programming (MILP) evolution models have been developed for the hydrogen infrastructure investment decisions to meet hydrogen demand for fuel cell vehicles.^12−16^ The aforementioned models include hydrogen production, storage, and distribution through road using a representative day per time period. Moreover, hydrogen transmission through pipelines has been studied using spatially explicit MILP frameworks.^17−19^

Industrial and mobility hydrogen markets were investigated using a multiobjective optimization approach including economic, environmental, and social aspects.^20,21^ Furthermore, an MILP model was developed for a green hydrogen supply chain to satisfy hydrogen industrial and maritime demand.^22^ The introduction of hydrogen in the heating sector was studied using spatiotemporal frameworks.^23,24^ Given the fact that heating demand has fluctuations during the day, hourly temporal resolution is incorporated for this case to determine both design and operational decisions.

Notwithstanding, the realization of a hydrogen economy by 2050 relies on several uncertain factors such as the cost and availability of hydrogen technologies, gas and electricity prices, and policy frameworks. Therefore, it is crucial to study the uncertainties related to hydrogen infrastructure planning to provide a viable energy transition strategy for low-carbon hydrogen investments.

Therefore, in the last decades, studies have focused on the introduction of uncertain parameters in hydrogen models using optimization-based methods. Table 1 provides an overview of hydrogen models with uncertainty considerations. As illustrated in Table 1, the majority of the works are formulated as MILP models, while flexible programming (FP) has also been employed.^25^ With respect to the temporal resolution, it is observed that most of the models are multiperiod (MP), while only a study considers multiple periods along with hourly resolution (H) within a day.

**Table 1:** Overview of Hydrogen Planning models under Uncertainty

	model type		uncertainty approach				spatial resolution	temporal resolution		uncertain parameters			
paper	MILP	FP	S	C	R	F		MP	H	D	C	F	A
Almansoori and Shah^27^	x		x				x	x		x			
Camara et al.^35^	x			x			x	x					x
Dayhim et al.^28^	x		x				x	x		x			
Fazli-Khalaf et al.^25^		x				x	x	x			x		
Hwangbo et al.^30^	x		x				x			x			
Kim et al.^26^	x		x				x			x			
Nunes et al.^29^	x		x				x	x		x			
Ochoa-Bique et al.^32^	x		x				x	x		x			
Robles et al.^31^	x					x	x	x		x			
Sabio et al.^36^	x		x				x	x			x	x	
Yang et al.^34^	x		x				x		x	x			x
Zhou et al.^33^	x				x		x	x	x	x			
proposed work	x		x				x	x	x	x	x	x	x

As indicated in Table 1, a significant portion of research focuses on the uncertainty of hydrogen demand^26−33^ (D). Besides demand, uncertain wind^34^ and primary energy sources availability^35^ (A) have been investigated, which are key elements in investment decisions. Moreover, there are inherent uncertainties in future hydrogen production cost estimates regarding technologies with a low readiness level. Thus, studies in the literature examined the role of uncertainty in operating costs^25^ (C). Additionally, the combination of different uncertain variables is explored, including demand, resources availability as well as feedstock (F) and technology costs, which further complicate decision-making in hydrogen investments.^34,36,37^

Concerning the uncertainty approach, most studies have employed a two-stage stochastic programming approach (S) to capture uncertainties^26,28−30,37^ while multistage stochastic programming has been also implemented by fewer works.^27,32,35^ Beyond stochastic programming, other optimization approaches have been utilized, including chance constrained programming^34^ (C), probabilistic fuzzy programming^25^ (F), and robust optimization^33^ (R).

Stochastic programming models are widely used in the literature to incorporate uncertainty.^38,39^ Although these models are computationally intensive due to the large number of equations and variables, advancements in computational capacity allow us to implement stochastic programming to process systems applications. To address the combinatorial complexity of stochastic programming, decomposition techniques are applied, such as Benders decomposition and Lagrangean relaxation.^38^

In this work, a multiperiod spatially explicit two-stage stochastic framework is developed for hydrogen infrastructure planning to meet residential, commercial, industrial, and transportation heating hydrogen demand. The model considers dual temporal resolution, including 10-year time steps and representative days for strategic and operational decisions, respectively. Uncertainty is incorporated in hydrogen demand, natural gas price, biomass availability, seasonality, and water electrolysis and wind and solar farm costs. The applicability of the model is demonstrated through a case study of heat demand in Great Britain (GB). The contributions of this paper focus on:

- The development of a two-stage stochastic multiperiod hydrogen optimization model with spatiotemporal resolution;
- The combination of different uncertainty parameters and the development of a scenario reduction framework to reduce combinatorial complexity;
- The investigation of uncertainty effect in decision-making regarding hydrogen infrastructure planning; and
- The impact of electrolytic hydrogen production on investment planning.

The remainder of the paper is structured as follows: The problem description is given in Section 2. The optimization framework is described in Section 3. A case study of hydrogen infrastructure planning in Great Britain is described in Section 4. Results analysis is provided in Section 5. Finally, Section 6 summarizes the concluding remarks.

## 2 Problem Description

### 2.1 Problem Statement

The goal of this work is the development of a two-stage stochastic programming framework for the optimal design of a hydrogen investment strategy over a given planning horizon. The proposed optimization framework aims to meet hydrogen demand and satisfy the Net-Zero CO_2_ emissions target. Moreover, it investigates the optimal infrastructure decisions in terms of type, capacity and location of hydrogen production and storage investments, capacity and location of H_2_ and CO_2_ pipelines, as well as the location of CO_2_ reservoirs. Regarding the operational decisions, the model aims to determine hydrogen production, storage, and transmission rates within a set of representative days.

Uncertainty is introduced in hydrogen demand, gas price, biomass availability, technology costs, and seasonality (renewables availability). First-stage (here-and-now) decisions, which are common in all of the scenarios, include the optimal location and capacity of production plants, storage sites as well as H_2_ and CO_2_ pipeline connections. All of the other variables, including operating decisions, are second-stage (wait-and-see) decisions, which are scenario-dependent. The overall problem statement can be summarized as follows. Given:

- Hydrogen demand and renewables availability hourly profiles in each region, time step, representative day, and scenario;
- Capital and operating costs for hydrogen production plants, hydrogen storage sites, renewable farms, and H_2_ and CO_2_ pipelines;
- Minimum and maximum capacity, ramp rates, and lifetime of production plants and storage sites;
- Minimum and maximum flow rate limits in pipelines;
- Capacity of hydrogen caverns and CO_2_ reservoirs;
- Hydrogen import price;
- Carbon tax and capture rates for CO_2_ emissions as well as CO_2_ emission targets for each time step;
- Biomass and land availability.

Determine the optimal:

- Capacity and location of production plants and storage sites;
- H_2_ production and storage rates in each region, time step, representative day, time slice, and scenario;
- H_2_ and CO_2_ pipeline investments between regions;
- H_2_ and CO_2_ flow rates between regions in each time step, representative day, time slice, and scenario;
- Electricity generation of renewable sources in each region, time step, representative day, and time slice;
- H_2_ import rates in each time step, representative day, and time slice.

So as to minimize the total system cost subject to CO_2_ emission targets, the key assumptions of the proposed model are summarized below:

- Hourly gas demand profiles are used as a proxy for industrial, residential, and commercial heating demand;^40^
- Transportation demand is distributed equally to the regions;
- Regional hydrogen transmission takes place through pipelines;
- Transmission distances are calculated as the distance between centroids of each region;
- Hydrogen pipeline connections are designed based on the layout of the incumbent gas pipeline network;
- Hydrogen distribution within a region is not considered;
- Electricity generated from renewable farms is used in water electrolysis units;
- Variable operating costs for renewable farms are not considered;
- Curtailment costs are not taken into account;
- Gaseous hydrogen is solely considered.

### 2.2 Spatiotemporal Resolution

**Figure 1:**
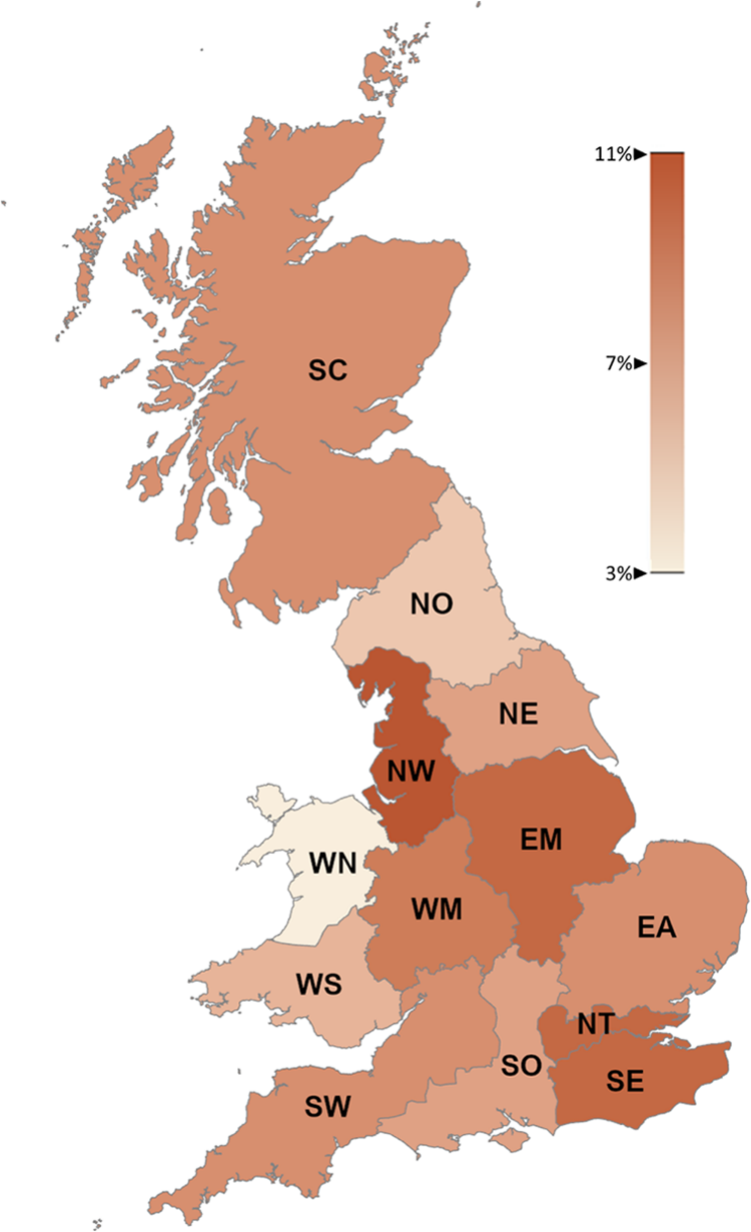
Hydrogen demand allocation in Great Britain.

Spatial resolution is a key feature of the model to capture regional differences regarding hydrogen demand, renewables, and land availability. Thus, GB is divided into 13 regions, as depicted in Figure 1, according to the local distribution zones (LDZ) of the incumbent gas network.^41^

**Figure 2:**
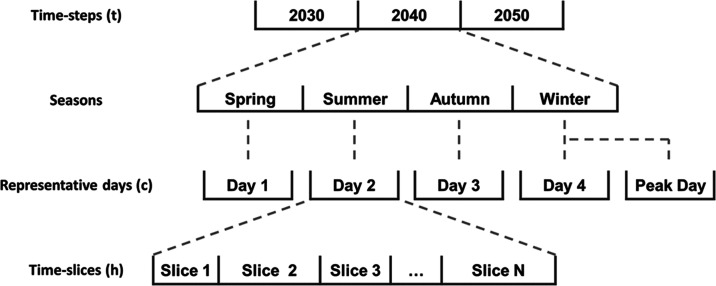
Temporal resolution of the model.

Concerning temporal resolution illustrated in Figure 2, the model considers three 10-year time steps (2030–2039, 2040–2049, and 2050–2059) in which investment decisions can take place. For each time step, the typical year is divided into 4 calendar seasons. Each season is represented by one typical day (cluster), which is selected using the *k*-medoids method based on real data hourly profiles for residential, commercial, and industrial demand as well as solar, wind onshore, and wind offshore availability. The *K*-medoids clustering method is employed as medoids (real data) can provide more accurate results due to higher fluctuations than centroids (average profiles).^42^ Operational decisions, such as production rate and flow rates between regions, are determined at the daily level. In this level, each day is divided into *N* time slices, which are clustered using an MILP model, as presented in Appendix B. Additionally, the peak demand day with hourly resolution is introduced to ensure the ability of the system to meet the high demand peaks.

### 2.3 Superstructure

**Figure 3:**
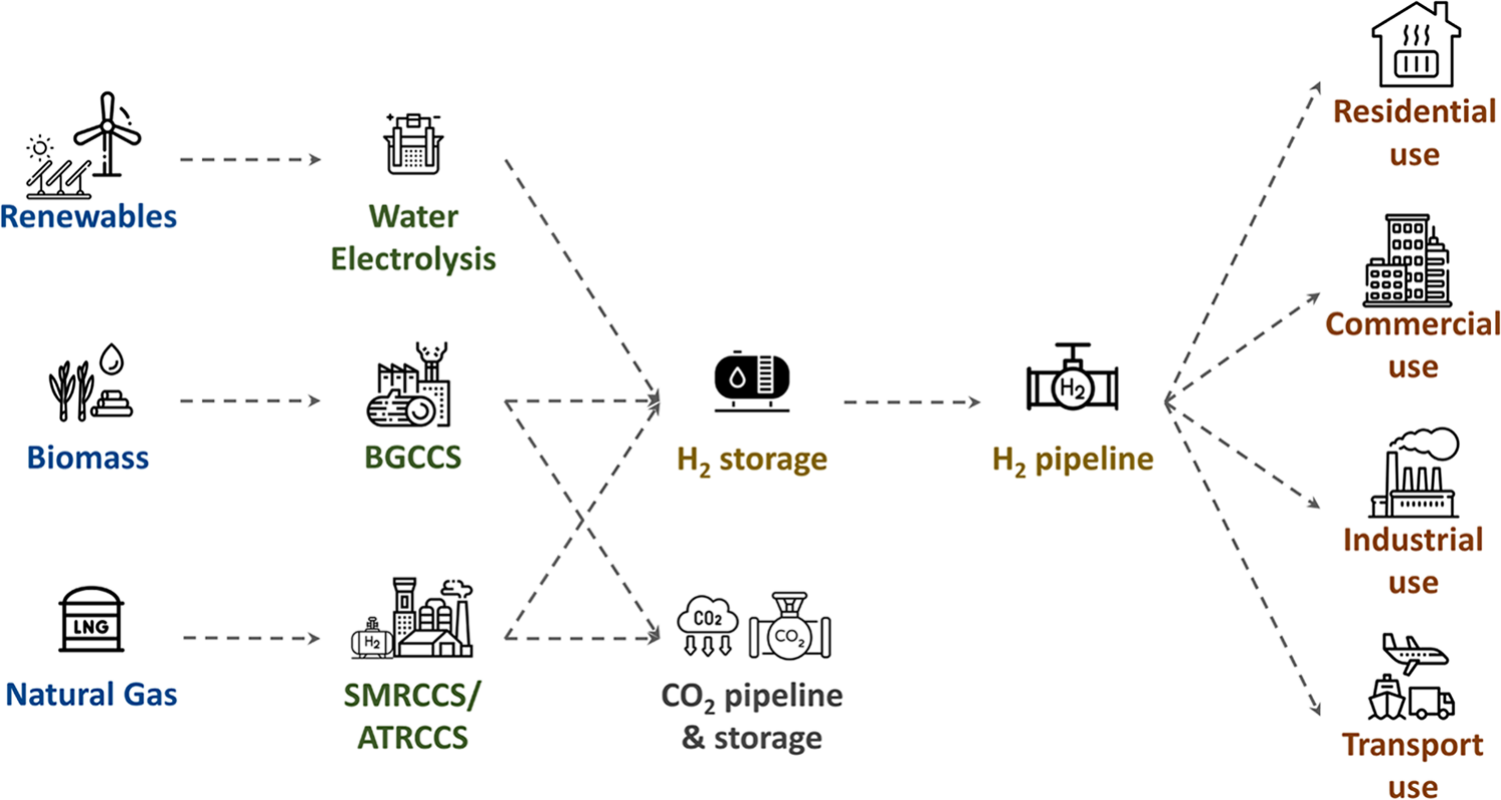
Hydrogen and CO_2_ superstructure.

This case study explores infrastructure investments regarding hydrogen production, storage, and transmission technologies, along with a carbon capture and storage (CCS) system, as depicted in Figure 3, to meet hydrogen residential, commercial, industrial, and transportation demand.

The considered hydrogen production technologies are steam methane reforming (SMR) with CCS, autothermal reforming (ATR) with CCS, biomass gasification (BG) with CCS, and water electrolysis (WE). Concerning water electrolysis, polymer electrolyte membrane (PEM) technology is employed due to its high operating density and reduced environmental impact.^43^ The electricity required for WE is generated from renewable technologies, including solar, onshore, and offshore wind farms.

Hydrogen storage is crucial for meeting future demands in the hydrogen economy. This study takes into account two types of storage vessels: high pressure storage vessel (HPSV) and medium pressure storage vessel (MSPV). Additionally, the installation of hydrogen storage caverns is considered. Hydrogen transmission between regions takes place through pipelines of 1 m diameter.

To minimize greenhouse gas emissions and environmental impact, SMR, ATR, and BG technologies are integrated with a CCS system. The CO_2_ emissions captured from the production units are transported to CO_2_ reservoirs via onshore and offshore pipelines with a diameter of 1.2 m. The CO_2_ reservoirs employed in this study can be established in the North and Irish Sea. More specifically, CO_2_ reservoirs are grouped into four GB offshore regions, including one in the East Irish Sea Basin and three in the North Sea (northern, central and southern).^44^

Comprehensive techno-economic data for the model were collected from various references and are available in our previous work.^45^

## 3 Optimization Framework

### 3.1 Mathematical Modeling

In this section, we present a summary of the mathematical formulation based on the model proposed in our previous work,^45^ which has now been modified as a two-stage stochastic multiperiod spatially explicit MILP model. A detailed description of the model constraints can be found in Appendix A.

In stochastic formulations, two kinds of optimization variables are considered: first-stage decision variables, which are common in all scenarios (here-and-now), and second-stage decision variables, which are different from scenario to scenario (wait-and-see). First-stage decisions are the investment decisions, including the optimal location and capacity of production plants, storage sites, renewable farms, as well as H_2_ and CO_2_ pipeline connections. All of the other variables constitute second-stage decisions, which are operational decisions (e.g., production rates, flow rate between regions, carbon emissions). This work incorporates uncertainties in different parameters, including hydrogen demand, renewables, and biomass availability as well as water electrolysis and solar and wind farm technologies costs.

#### 3.1.1 Total System Cost

The objective of the model is the minimization of the total system cost (TSC), which is a summation of the first-stage cost *(*TC^F^*)* and second-stage cost (TC_k_^S^) in each scenario *k* multiplied by the probability (pb_*k*_) of the occurrence of each scenario *k*, as presented in eq 1.
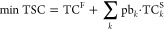
1

The first-stage total cost (TC^F^) consists of the storage capital cost (SCC), pipeline capital cost (PLCC), and operational cost (PLOC_k_), as shown in eq 2. The second-stage total cost (TC_k_^S^) consists of the production capital cost (PCC_k_), production operational cost (POC_k_), storage operational cost (SOC_k_), carbon emissions cost (CEC_k_), hydrogen import costs (IIC_k_), renewables cost (ReC_k_), biomass (BC_k_), and natural gas (NGC_k_) costs, as presented in eq 3.
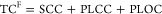
2
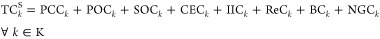
3A detailed description of system costs can be found in Appendix A in eq A1 (A11).

#### 3.1.2 Mass and Energy Balances

Hydrogen energy balance is described by eq 4. More specifically, in each region *g*, time step *t*, representative day (cluster) *c* and time slice *h*, the total production rate (Pr_pgtchk_), the flow rate (*Q*_g′gtchk_) to region g, the rejected hydrogen from storage site s and the imported hydrogen (Imp_gtchk_) are equal to the flow rate (*Q*_gg′tchk_) from region g, the injected hydrogen to storage sites s (*Q*_gstchk_^I^), and the total demand (TD_gtchk_).
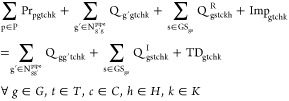
4The mass balance of CO_2_ is expressed by eq 5. The left-hand side represents the onshore CO_2_ flow rates to region *g* from other regions *g*′ (Q_g′gtchk_) and the captured CO_2_, which is equal to the hydrogen production rate (Pr_pgtch_) multiplied by a coefficient of CO_2_ capture for each production technology type (y_pt_^c^). The right-hand side represents the onshore CO_2_ flow rate from region *g* to other regions *g*′ (Q_gg′tchk_) and the offshore CO_2_ flow rate () from region *g* to reservoir *r*.
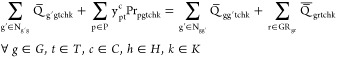
5

#### 3.1.3 Capacity Expansion

Capacity expansion decisions are here-and-now, as infrastructure investments are common for all scenarios. The production plants availability is defined by eq 6.
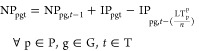
6where NP_pgt_ stands for the available production plants, IP_pgt_ stands for the newly invested plants, and LT_p_^P^ is the lifetime of production technology p. Storage site availability is presented in eq 7.
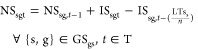
7where NS_sgt_ and IS_sgt_ are the available and the new invested storage sites, respectively, while LTs_s_ is the lifetime of storage technology *s*, and *n* is the duration of each time step.

Pipeline availability for hydrogen transmission between regions and to the storage caverns as well as for onshore and offshore CO_2_ transmission are defined by eqs 8–11.
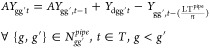
8
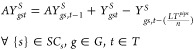
9
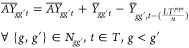
10
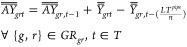
11where *AY*_gg′Pt_ and *Y*_gg′t_ represent the available and newly commissioned hydrogen pipeline connections between regions, respectively. *AY*_gst_^S^ and *Y*_gst_^S^ are the available and new pipeline connections for the underground storage caverns. Additionally, *AY*_gg′t_, *Y*_gg′t_, , and  are the available and the newly invested pipeline connections for onshore and offshore CO_2_ transmission.

#### 3.1.4 Storage Constraints

**Figure 4:**
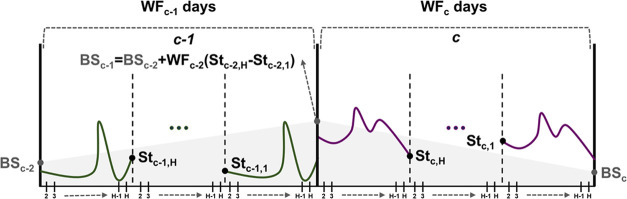
Storage modeling visual representation.

To represent storage within a time step *t*, the daily (intraday) storage profile and the accumulated (interseasonal) storage between the seasons are modeled, as illustrated in Figure 4.

The intraday storage rate (St_sgtchk_) is equal to the storage rate in the previous time slice and the hydrogen that is injected, minus the hydrogen which is withdrawn in each storage site *s*, region *g*, time step *t*, cluster *c*, and time slice *h.* St^init^ is the initial storage rate in the first time slice. *Q*_gstch_^*I*^ and *Q*_sgtch_^*R*^ stand for injected and retrieved hydrogen from storage type *s*.
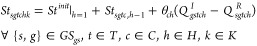
12where θ_ch_ denotes the number of hours in a cluster *c* and time slice *h*. The interseasonal storage rate BS_sgtck_ is calculated for each cluster *c* separately according to eq 13.
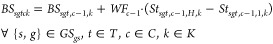
13where WF_c_ is the weight of cluster *c* and *H* stands for the last time slice of each cluster *c*. Total storage rate is limited by lower and upper bounds, as defined in eq 14.
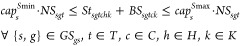
14where cap_s_^min^ and cap_s_^S_max_^ are the minimum and maximum storage rates, respectively, while NS_sgt_ is the number of available storage sites of technology *s*, region *g*, and time step *t*.

#### 3.1.5 Emissions Target

An emission target (et_t_) for hydrogen production is considered in the model, as defined by eq 15.
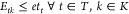
15where *E*_tk_ stands for the total CO_2_ emissions from hydrogen production in time step *t* and scenario *k*. For this case study, the emission target is focused exclusively on 2050, with the aim of achieving Net-Zero total emissions.

#### 3.1.6 WE Penetration

Water electrolysis is a key element to achieve a green transition in the energy mix. To this end, U.K. hydrogen strategy policy forecasts an increase in electrolytic hydrogen production.^46^ Therefore, eq 16 enforces that at least a β percentage of the total average production is from water electrolysis.
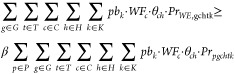
16It is worth noting that β is equal to 0 for the base case. Apart from the aforementioned equations, the mathematical model includes hydrogen production, storage, transmission, and import constraints as well as electricity production, CO_2_ emissions, and CO_2_ reservoir constraints. The proposed framework is formulated as an MILP model and aims to minimize the total cost subject to eqs 1–16 and (A1–AA30).

### 3.2 Solution Scheme

The mathematical framework considers spatiotemporal resolution as well as uncertainty in several parameters (costs, availability, demand), resulting in a computationally intensive model. Thus, a hierarchical approach is used to tackle the combinatorial complexity, achieving cost-efficient design decisions in less computational time, as described in detail in our previous work.^45^ The hierarchical solution approach can be described in the following steps:

- Solve the model without considering the H_2_ and CO_2_ pipeline network decisions;
- Determine the production (NP_pgt_) and storage (NS_sgt_) investment decisions;
- Fix NP_pgt_ and NS_sgt_ decisions for all time steps;
- Solve the reduced model;
- Determine the H_2_ and CO_2_ pipeline design and all continuous variables.

eq 8–11, (A5, A6), and (A17–A19) are not included in the first step as they are related to pipeline costs, availability, and maximum flow rate. Thus, the number of discrete variables decreases, which reduces the complexity. The feasibility of the model regarding pipeline network is ensured by setting an upper bound in the regional flow rates, which is equal to the maximum flow.

Future hydrogen network design involves many uncertainties related to the costs and demands of the energy systems. The proposed model incorporates uncertainty in hydrogen demand, natural gas price, techno-economic data, biomass, and renewables availability. It is assumed that the scenarios are equal to all of the possible combinations of realizations of the uncertain parameters. Thus, a large number of scenarios are obtained, which makes the model computationally prohibitive.

To deal with combinatorial complexity, scenario reduction is essential. Using a scenario reduction method, the cardinality of scenarios decreases while trying to keep the information on the full set in the reduced one.^47^ The concept of scenario reduction was introduced by ref (48), which uses a probability metric to obtain the closest subset from a larger scenario set. GAMS–SCENRED^49^ is a popular software using probabilistic information apdeteplied in stochastic programming approaches.^50−53^

**Figure 5:**
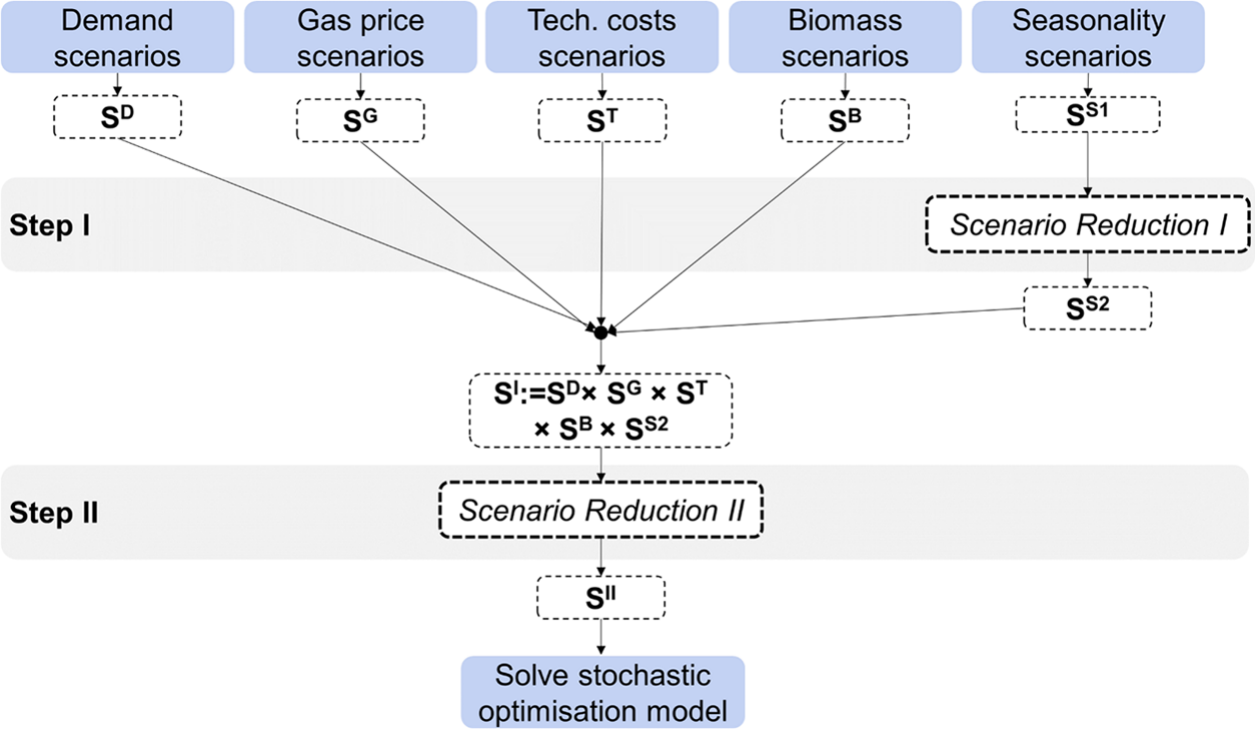
Scenario reduction steps.

The suggested framework consists of two steps, and it is illustrated in Figure 5. The first reduction step (Step I) is performed on seasonality scenarios, decreasing the number from an initial set *S*^*S*1^ to a reduced set *S*^*S*2^. This step is employed, as it is observed that a similar number of scenarios need to be used for the different parameters in the second step. Assuming initial sets of demand scenarios *S*^*D*^, gas price scenarios *S*^*G*^, technologies cost scenarios *S*^*T*^, and biomass scenarios *S*^*B*^, the second reduction step (step II) is performed on the combination of the aforementioned sets and the reduced set *S*^*S*2^. Both steps are conducted through fast forward selection using GAMS–SCENRED. The number of scenarios in the reduced set *S*^*S*2^ is determined using a user-defined threshold: the marginal relative probability distance metric (mRPD).^50^ Mathematical description of mRPD can be found in Appendix C. Additionally, the computational time of the optimization model is employed as an indicator for scenario number selection, as presented in Section 5.1.

## 4 Case Study

The two-stage stochastic MILP framework investigates the optimal infrastructure planning regarding hydrogen production, storage, and transmission in Great Britain. The model aims to satisfy uncertain hydrogen residential, commercial, industrial, and transportation demand, while uncertainty is also introduced in biomass availability, gas price, technology costs, and seasonal renewable sources availability. In this section, the uncertain parameters are described while the detailed techno-economical data used in this analysis can be obtained from the Supporting Information of our previous work.^45^

### 4.1 Hydrogen Demand

The hydrogen roadmap in the U.K. forecasts a significant increase in hydrogen demand to decarbonize industry and provide a greener transition in transport, residential, and commercial heat sectors.^11^

There is high uncertainty about the precise role of hydrogen in the future energy mix and related policies and regulations. According to the analysis of the Sixth Carbon Budget,^54^ around 200–460 TWh of hydrogen could be needed in 2050. Thus, the demand constitutes a highly uncertain parameter.

Total demand consists of residential, commercial, industrial, and transport demand. Spatially explicit historical hourly gas profile data are obtained for residential, commercial, and industrial sectors. Hydrogen profiles are adjusted as a penetration to the gas profiles to provide a realistic demand, capturing seasonality and peaks.

To this end, in the present work, hydrogen demand scenarios (*S*^D^ = 5) are considered, with a total demand range from 205 to 465 TWh in 2050. Scenarios 1 and 2 (30% probability each) are obtained from National Grid ESO demand scenarios,^55^ while scenarios 3–5 (with probabilities 10, 20, and 10%) are calculated as a penetration of 25, 50, and 75% in the historical natural gas demand, respectively.^40^ Detailed data for the demand are presented in Appendix D.

### 4.2 Biomass Availability

Biomass gasification coupled with CCS is a key element technology due to the resulting negative emissions, which are vital to achieve Net-Zero goal. However, biomass availability is limited, which constitutes an additional uncertain parameter of the current work.

In this case study, nonwaste biomass feedstock is considered, consisting of agricultural residues, forestry residues, and energy crops. Different scenarios for future U.K. nonwaste biomass production are explored by the Department for Energy Security and Net-Zero in the U.K.^56^ based on existing policies and evidence. Two illustrative scenarios (ambitious and restricted) are included in the aforementioned analysis, in which the differences are based on future land use and policies around cultivation. These two scenarios are considered as the low and high scenarios (with a probability of 30% each), while a base scenario (with a probability of 40%) is introduced as the mean case (*S*^B^ = 3). An assumption of 50% availability of the nonwaste biomass for gasification technologies is made as biomass can be used as feedstock for other processes such as power generation, methane gas production, etc. Biomass availability for each scenario can be found in Appendix D.

The available biomass feedstock (energy crops, agricultural residues, forestry) is discretized in the 13 regions of Great Britain according to local gas distribution zones (LDZ). Biomass transportation between the regions is not taken into account in this work. Therefore, the availability of biomass sources in each region is calculated using a geographically distributed data analysis.^57^

### 4.3 Gas Price

Future gas prices play a significant role in hydrogen investment strategic decisions,^45^ as gas is used as feedstock in reforming technologies. However, forecasting the natural gas price constitutes a challenge due to the many factors influencing gas markets.

To this end, an analysis of historical gas prices is conducted, and forecast scenarios (*S*^G^ = 5) up to 2050 are obtained using an empirical distribution function based on natural gas forecasts of National Grid ESO.^55^ The probabilities for scenarios 1–5 are 26.8, 62.5, 9.7, 0.7, and 0.3%, respectively. The prices of each scenario are listed in Appendix D.

### 4.4 Technology Cost

Another parameter which is crucial for infrastructure planning is technologies cost. Thus, with regard to less mature technologies, there is an inherent uncertainty in capital and operating costs. In this context, uncertainty is introduced in water electrolysis capital and operational costs as well as in capital costs in solar, wind onshore, and offshore farms.

For all of the technologies, we assume low, base, and high-cost scenarios (*S*^C^ = 3) according to data obtained from the Department for Energy Security and Net-Zero in U.K.^58,59^ Probabilities assigned are 25, 50, and 25% for low, base, and high scenarios, respectively. Detailed data for the costs are described in Appendix D.

### 4.5 Seasons

As described in Section 2.2, a typical day for each season is employed to capture different demand and renewable availability fluctuations. Uncertainty is introduced in the selection of the typical day to increase the fidelity of the model.

In this context, *k*-medoids clustering is used to select three typical days in each season and assign the probability. The hourly profiles, which are clustered, include solar wind onshore and wind offshore availability as well as residential, commercial, and industrial demand profiles for each LDZ region.

After the generation of the typical days for each season, it is assumed that all possible combinations between seasons are allowed, resulting in an initial set of *S*^S1^ = 81 scenarios. As depicted in Figure 5, after scenario reduction in Step I, the number of scenarios is decreased to *S*^S2^ = 5, which is used in the scenario reduction in Step II.

## 5 Computational Results and Discussion

This section demonstrates the applicability of the proposed framework through the implementation described in Section 4. Section 5.1 focuses on scenario reduction. Section 5.2 presents the base case results, while Section 5.3 focuses on the penetration of WE in the hydrogen mix.

The computational runs were performed on an Intel Core i9–10980XE CPU operating at 3.00 GHz with 128 GB of RAM, using GAMS 46.1.0^60^ and Gurobi 11.0.0^61^ solver with default options. The machine has 18 cores, while 30 threads are used in each optimization. Termination criteria for each optimization are set to 24 h CPU time-limit or 5% relative optimality gap for each step. Upper bounds of 50 and 80 are employed for IP_pgt_ and IS_sgt_ variables, respectively.

### 5.1 Scenario Reduction

The number of selected scenarios constitutes a crucial trade-off decision. A larger number of scenarios provide a more accurate representation of the uncertainty space, thereby enhancing decision-making. On the other hand, as the number of scenarios increases, the combinatorial complexity of the model grows exponentially, making it intractable to solve.

An initial set of 1125 scenarios is generated as described in Section 4, while scenario reduction is conducted following the procedure described in Section 3.2. Marginal relative probability distance (mPRD) metric and computational time are used to select the number of scenarios in the reduced set. Mathematical definitions for mRPD can be found in Appendix C.

**Figure 6:**
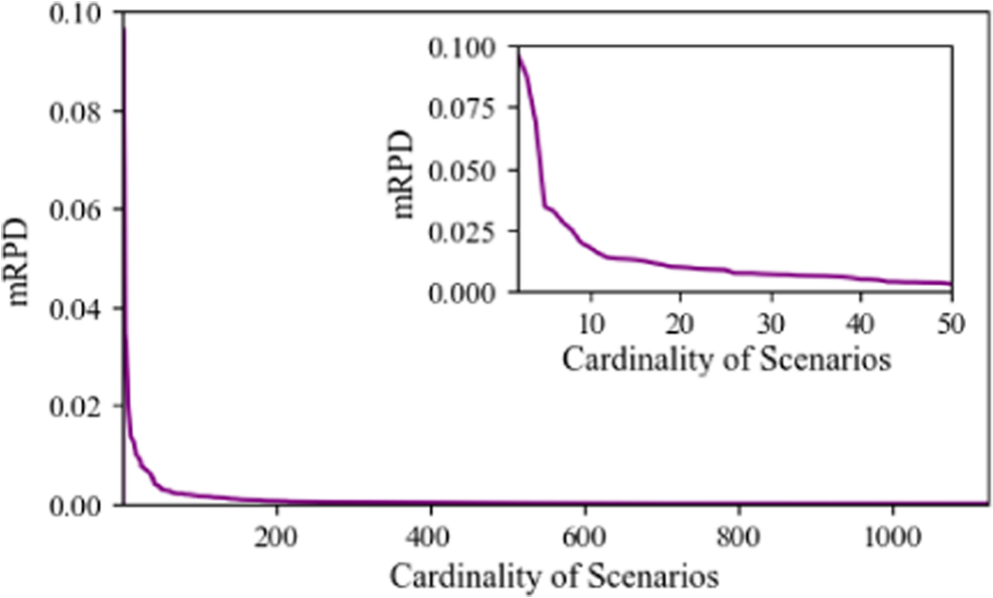
mRPD for different cardinalities of reduced scenario sets from Step II reduction.

Figure 6 shows how the marginal relative probability density (mRPD) decreases as the number of selected scenarios increases. The mRPD is expected to decrease monotonically with an increase in the number of scenarios. Thus, using a stopping criterion based on mRPD allows us to retain only those scenarios that reduce the relative probability distance by at least a certain threshold. Although this threshold is somewhat subjective, it provides a clearer method for balancing information accuracy with the number of scenarios.

Moreover, an exponential increase in computational time of the proposed two-stage optimization model is observed as the number of selected scenarios grows. In the case of 15 scenarios, the computational time is about 5 h, while for 25 scenarios, it increases to approximately 31 h. This sharp increase in CPU time makes the model intractable beyond 25 scenarios. Therefore, computational time becomes a crucial factor in determining the size of the reduced scenario set.

In this case, an approximate value of 1% for mRPD is used, identified as an elbow point. Additionally, sensitivity analysis shows that the model becomes computationally intractable with more than 20–25 scenarios, depending on the case study. Based on these criteria, 25 scenarios are selected as the base case for the reduced scenario set, while 20 scenarios are implemented for WE penetration cases. A more detailed sensitivity analysis is presented in Section 5.2.

### 5.2 Base Case

Within this section, a comprehensive analysis of the basic case is presented. Initially, the time slices within a day are defined, establishing the temporal framework. Following this, the number of scenarios is determined, evaluating the impact in the computational results. Finally, computational results are discussed along with a comparison between deterministic and stochastic programming approaches.

To capture the temporal variability in the system, the number of time slices within a day needs to be defined. 24 hourly time slices constitute the most widely used approximation for the operating decisions. However, this increases significantly the equations and variable number, affecting the computational complexity of the model and making it intractable.

**Figure 7:**
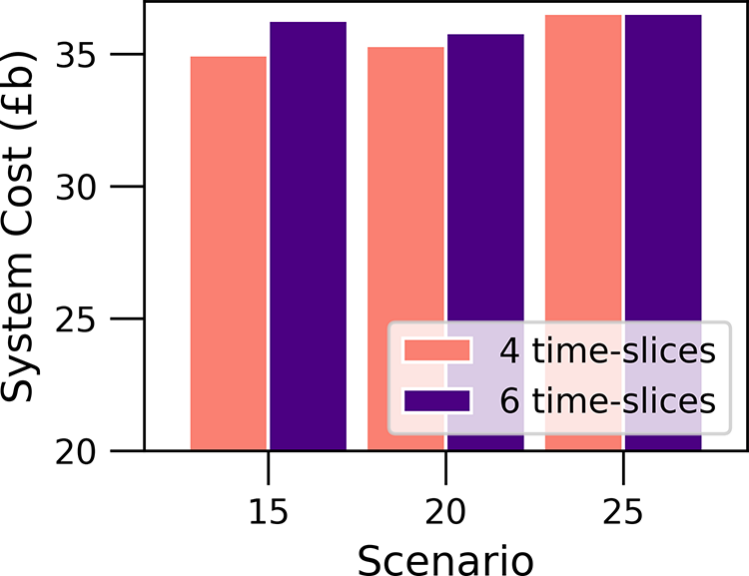
System cost for 4 and 6 time slices for different numbers of scenarios

To this end, each representative day is divided into 4 and 6 time slices using an MILP model, as presented in Appendix B. The effect of the number of time slices for different scenario numbers is illustrated in Figure 7. While the 4 time slice model results in a slightly less expensive system compared to the 6 time slice case, this difference can be attributed to the lower temporal resolution. However, the 6 time slice model offers a more detailed and accurate representation of daily fluctuations in demand and renewable availability, leading to a better approximation of the system.

To assess the quality of the solutions across the different number of time slices, system cost (SC) is compared with the expected system cost (ESC). ESC is derived by enforcing the first-stage variables obtained from each solution to the initial scenario set (1125 scenarios) with 24 h time slice resolution. This comparison enables an evaluation of how closely the time slice approximations reflect the results of hourly resolution modeling. For the 25 scenarios, where SC is approximately equal for 4 and 6 time slices, the ESC for the 4 time slice configuration is 1% higher, as presented in Table 2. This observation suggests that despite the higher system costs, the finer granularity of 6 time slices provides more accurate insights into system behavior.

**Table 2:** System Cost (SC) and Expected System Cost (ESC) of 25 Scenarios for Different Time slices

number of time slices	4	6
SC	36.5	36.5
ESC	37.6	37.2

**Figure 8:**
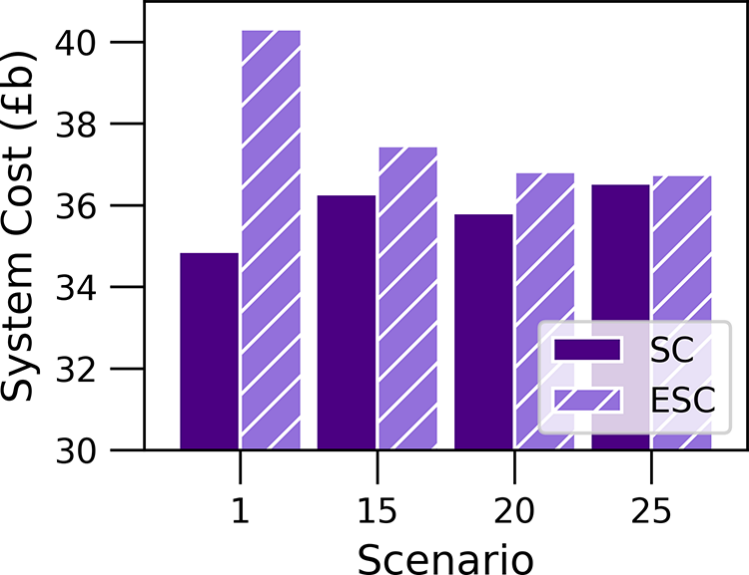
System cost (SC) and expected system cost (ESC) for a different number of scenarios.

An analysis for a different number of scenarios is carried out to investigate the impact of the deterministic (i.e., one scenario) and stochastic programming approaches on the computational results. Figure 8 depicts SC and ESC for different numbers of scenarios. ESC is calculated from the enforcement of first-stage variables in the initial scenario set (1125 scenarios) so as to test the quality of solutions. The deterministic approach (1 scenario case) includes the base case for all of the uncertain parameters, which has the biggest probability. On the other hand, the stochastic approach includes a number of scenarios that result from scenario reduction, as described in 3.2. For the 1 scenario case, the ESC increase compared to the corresponding SC is equal to 15.6%. As the scenario number increases, this value decreases, reaching 0.6% for 25 scenarios. Thus, taking into account also the analysis from Section 5.1, 25 scenarios are selected for the base case study.

**Figure 9:**
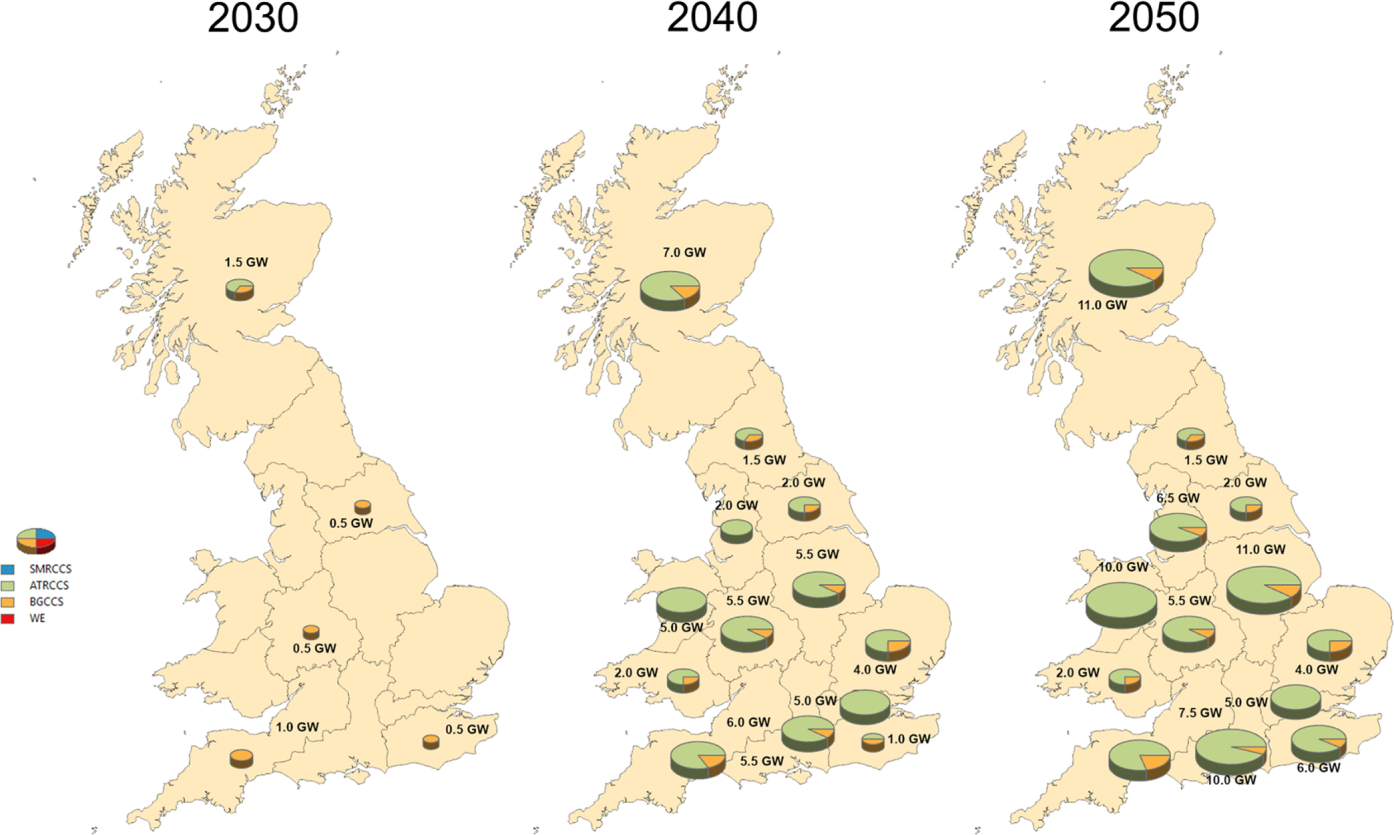
Production capacity maps.

The optimal infrastructure design of hydrogen production plants in Great Britain to meet the hydrogen demand is shown in Figure 9. A total of 4 GW is installed, consisting of ATR CCS and BG CCS technologies, with key locations in Scotland, the West Midlands, North East, South East, and South West England. Capacity expands significantly to 52 GW by 2040 and 82 GW by 2050, with production units located in all GB regions. ATR CCS technology dominates the production mix due to its cost efficiency and reduced CO_2_ emissions. Additionally, the BG CCS plays an important role in further lowering overall net emissions. SMR CCS is not included in the optimal design, as despite its lower capital cost compared to ATR CCS, it falls short in delivering the same level of cost efficiency and emissions reductions.

**Figure 10:**
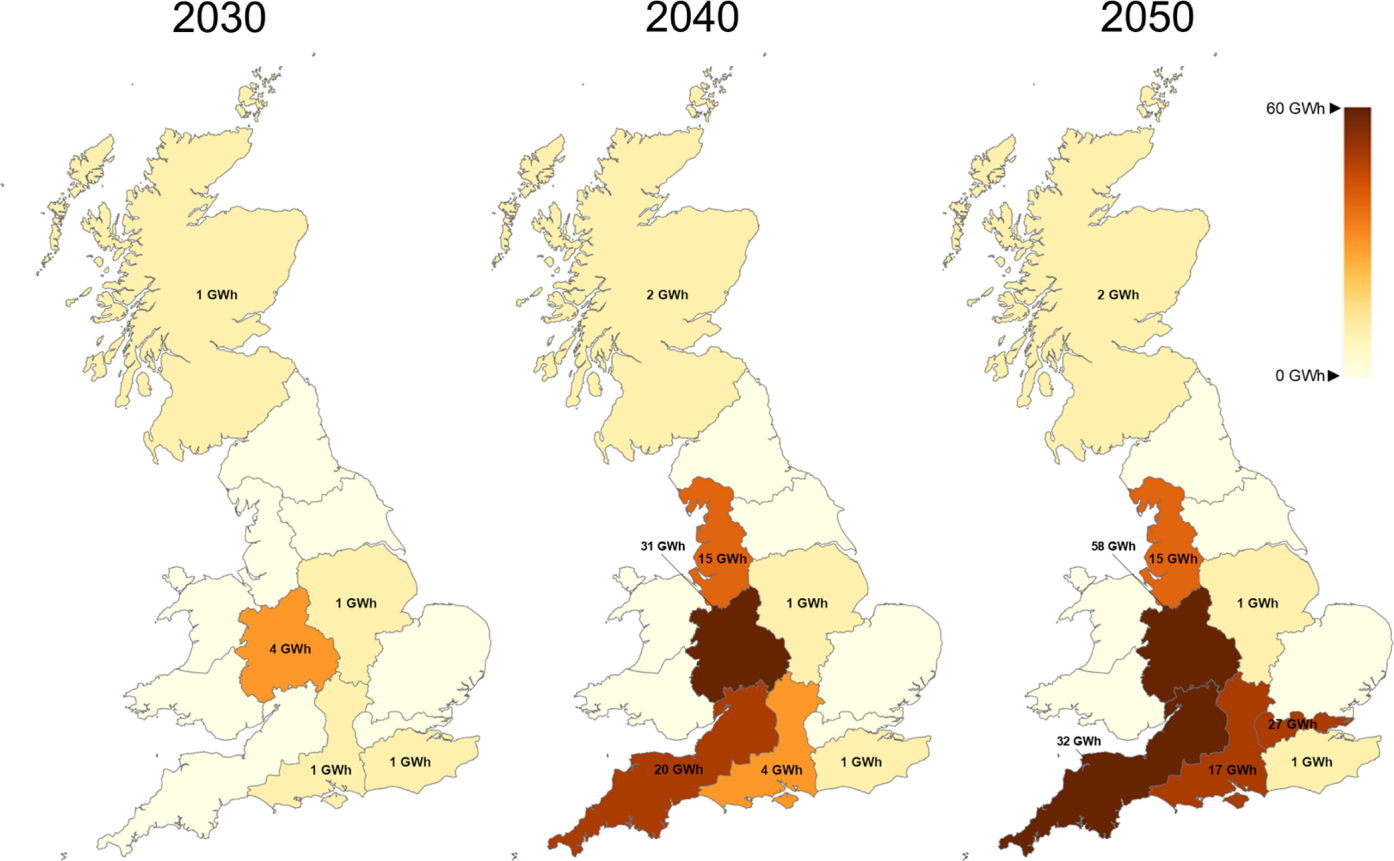
Storage capacity maps.

Figure 10 illustrates hydrogen storage maps from 2030 to 2050. Storage is a key element of a hydrogen network, ensuring the security and reliability of the hydrogen network. By 2030, 8 GWh of storage capacity of pressure vessels is installed, located with key facilities in Scotland and Central/South England. As hydrogen demand grows, storage requirements increase, reaching 74 GWh mostly located in West England. Finally, by 2050, total hydrogen storage capacity rises to 153 GWh while 58 GWh are installed in the West Midlands and 32 GWh in the South West. This expanded storage infrastructure is critical to maintain supply security, manage seasonal variations in demand, and support the broader energy transition.

**Figure 11:**
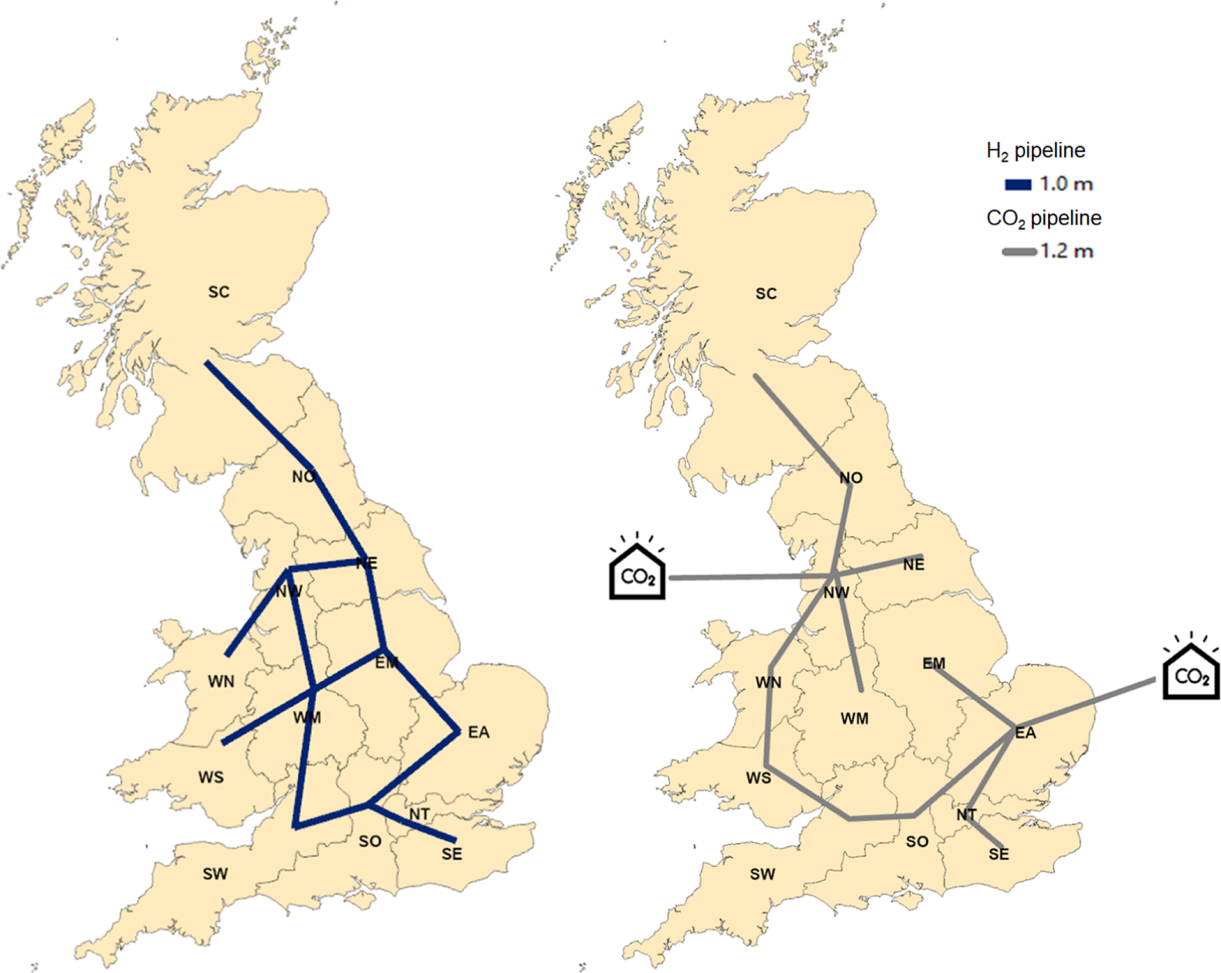
H_2_ and CO_2_ pipeline network in 2050.

Hydrogen transmission between regions is facilitated by pipeline networks. As shown in Figure 11, the hydrogen pipeline network will connect most Great Britain regions by 2050. This network will be critical for establishing a fully integrated infrastructure to transport low-carbon hydrogen across GB.

To support CCS in low-carbon hydrogen production, CO_2_ pipelines are established, as illustrated in Figure 11. In addition, two major CO_2_ storage reservoirs are established, one located in the southern North Sea and the other in the East Irish Sea Basin. These reservoirs serve as key hubs for storing captured carbon, playing a crucial role in reducing emissions across the system.

In Table 3, the MILP model size and computational performance of the base case are summarized. The total computational time is 31 h, while an optimality gap below 3% is achieved in both steps of the hierarchical approach. Monolithic approach results underscore the significance of model decomposition as it achieves a 49% larger objective function in 48 h.

**Table 3:** Base Case Model Size and Computational Performance

	hierarchical		
approach	Step 1	Step 2	monolithic
continuous variables	1,067,474	1,067,474	1,067,474
discrete variables	246	384	384
equations	1,410,273	1,697,889	1,697,889
computational time (h)	15.6	15.4	48.0
optimality gap (%)	1.42	2.72	39.10
objective function (£b)	36.5		54.4

**Figure 12:**
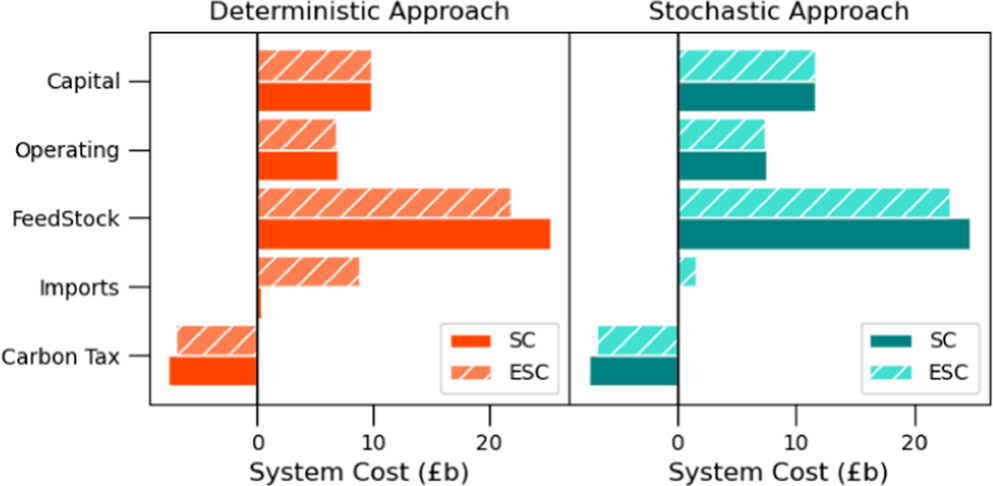
Cost breakdown.

To evaluate the quality of stochastic programming approach, a comparison with the deterministic approach is carried out. Figure 12 provides a detailed breakdown of the system costs alongside the expected system costs for the two approaches. It can be observed that feedstock costs are overestimated by 8% in the stochastic approach and by 16% in the deterministic approach when compared with ESC values due to significant fluctuations in demand scenarios. Additionally, Figure 12 highlights the substantial increase in import costs. More specifically, in the stochastic case, import costs rise from a SC of £b 0.12 to an ESC of £b 1.56, while the increase is more significant in the deterministic case, jumping from £b 0.43 to £b 8.79. This indicates that the deterministic approach may lead to suboptimal decisions, driving a higher reliance on imports and significantly elevating overall system costs.

**Figure 13:**
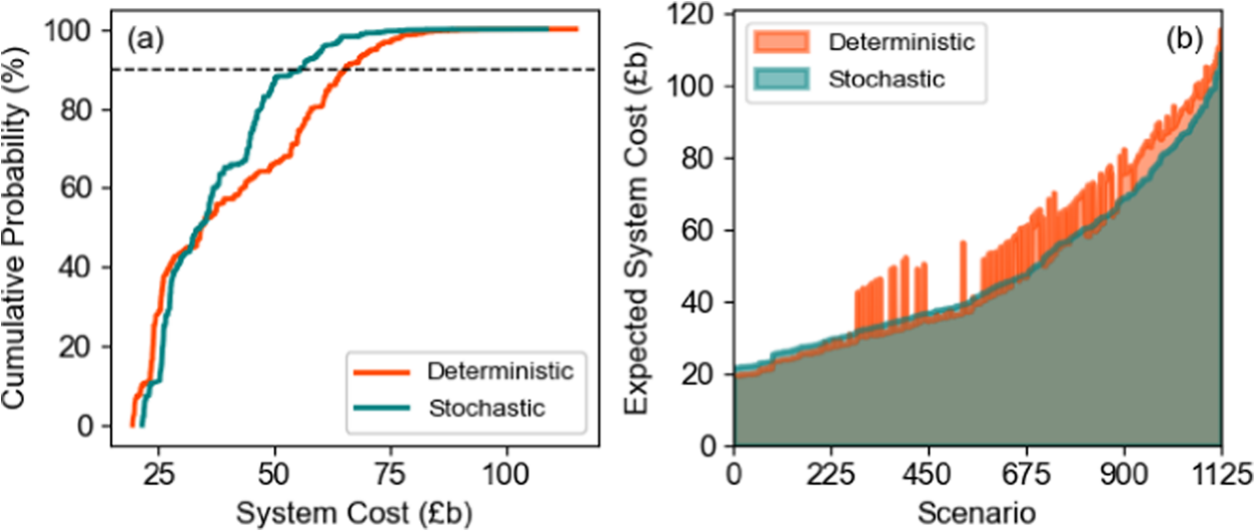
(a) Probability of system cost. (b) Expected system cost of scenarios.

Figure 13a demonstrates that while the deterministic approach offers slightly better outcomes in low-cost scenarios, the stochastic solution results in a narrower distribution of ESC. Furthermore, it is shown that 90% of scenarios provide a solution lower than £b 55 for the stochastic case compared to £b 65 for the deterministic one. This suggests that in the majority of scenarios, the stochastic approach delivers a more cost-effective solution, as illustrated also in Figure 13b. Thus, by incorporating uncertainties, the stochastic approach provides a more risk-neutral and balanced strategy, which leads to a more sustainable and cost-effective pathway to meet hydrogen demand.

### 5.3 Water Electrolysis Penetration

The U.K. government has set goals for hydrogen production capacity, with a commitment that water electrolysis will be included in the production mix.^46^ This focus on electrolytic hydrogen production underscores the U.K.’s commitment to developing low-carbon hydrogen pathways, leveraging renewable electricity to produce clean hydrogen through electrolysis. Achieving this target is expected to play a critical role in the country’s broader decarbonization efforts.

As discussed in Section 5.2, it can be concluded that ATR and BG technologies coupled with CCS constitute the most cost-efficient options for a low-carbon hydrogen strategy. However, the U.K. government and policymakers incorporate water electrolysis, which relies on renewable electricity, into their hydrogen strategy due to its environmental advantages as a greener option. Therefore, this section focuses on different electrolysis penetration cases to investigate different infrastructure strategies and the impact of uncertainty.

Thus, a constraint is imposed requiring that a certain percentage of hydrogen production must come from electrolysis. Two cases are examined: 10 and 20% electrolysis penetration. For this analysis, 20 scenarios are considered for the base and WE penetration cases, as a larger number of scenarios would render the model computationally intractable for WE cases.

**Figure 14:**
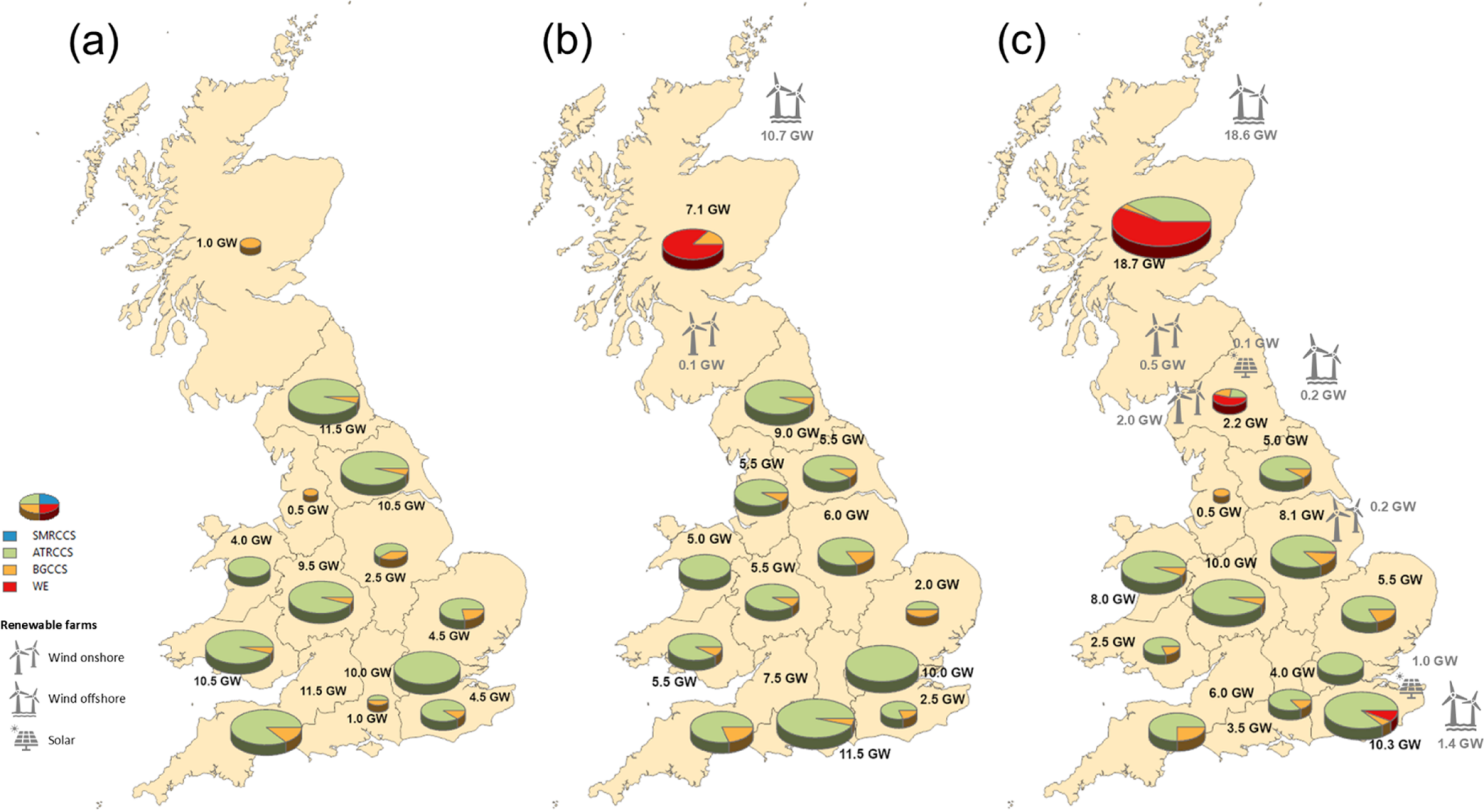
Production capacity maps (a) without any penetration constraint, (b) 10% penetration, and (c) 20% penetration in 2050.

A comparison of the three cases in terms of the production capacity mix is illustrated in Figure 14 for 2050. It is observed that there is a slight increase in the total production capacity, as the penetration of electrolytic hydrogen rises from 81.5 GW in the base case to 82.6 GW and 84.3 GW for 10 and 20% penetration, respectively. Notably, production capacity in Scotland exhibits a significant increase with higher electrolysis penetration. In the 10% case, 7.1 GW are installed, of which 6.7 GW are dedicated to electrolysis and 0.5 GW to BG CCS. In the 20% case, investments reach 18.7 GW, including 10.7 GW of electrolysis, 7.5 GW of ATR CCS, and 0.5 GW of BG CCS. This expansion can be attributed to Scotland’s abundant wind resources, as both onshore and offshore wind farms are established to power electrolysis in both scenarios. For the 20% case, WE plants are located also in the east Midlands, northern and southeast England, with solar, wind onshore, and offshore farms generating the required electricity.

**Figure 15:**
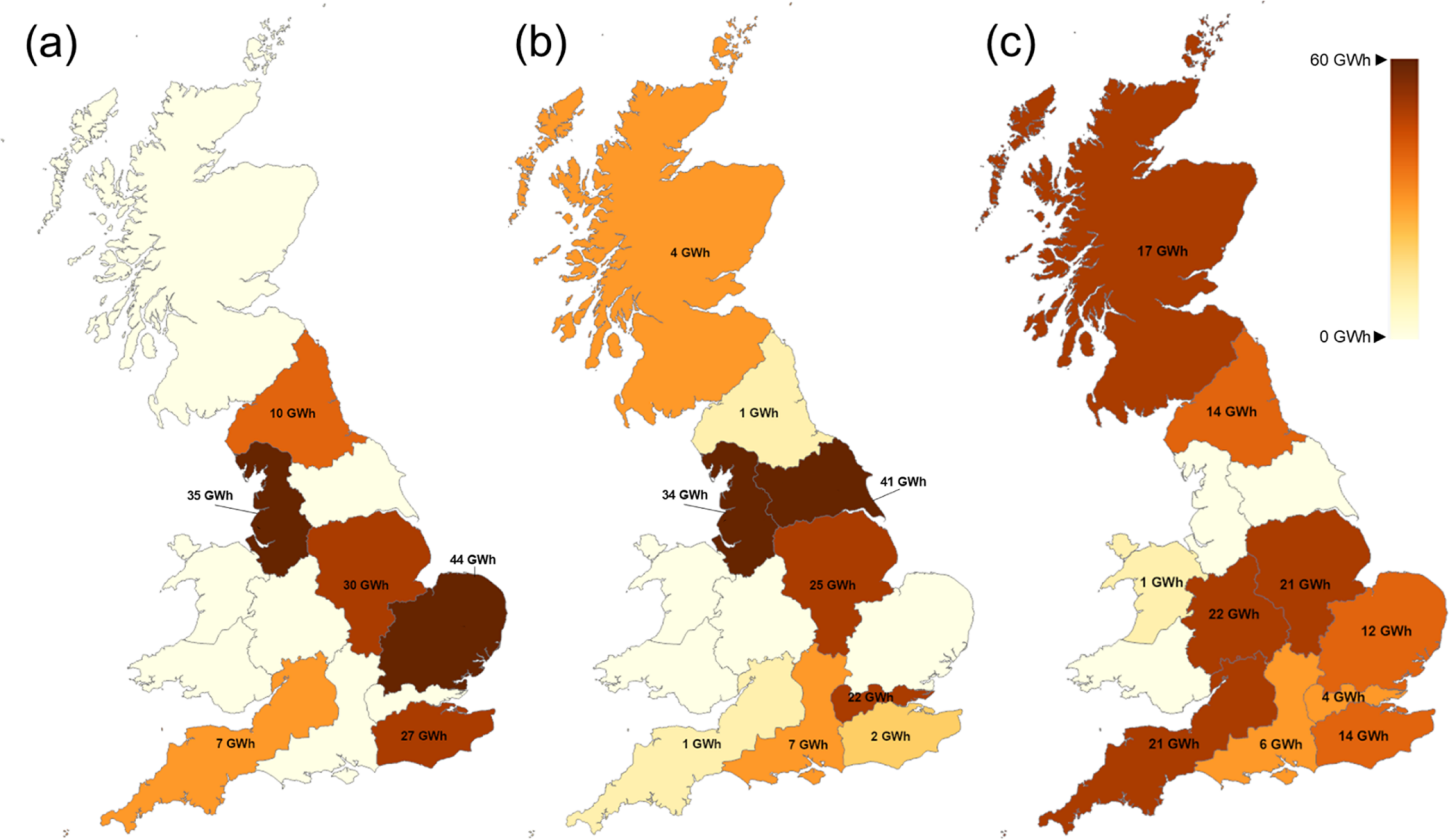
Storage capacity maps (a) without any penetration constraint, (b) 10% penetration, and (c) 20% penetration.

Storage allocation is depicted in Figure 15 for the three different WE penetration cases. In contrast to production, storage capacity decreases when WE penetration increases. Base case optimal design suggests a total of 153 GWh storage capacity while 137 and 132 GWh are invested in 10 and 20% of cases, respectively. Moreover, it can be observed that in 20% of cases, capacity is decentralized with investments in most of the regions, both in northern and southern GB. On the other hand, in the base case in which no electrolytic hydrogen is produced, storage capacity is located mostly in the center of GB. This centralization of storage highlights a more localized approach to meet demand in the absence of WE. However, the decentralized storage may better support regional flexibility and system resilience, given the fact that electrolytic hydrogen depends on renewables availability.

**Figure 16:**
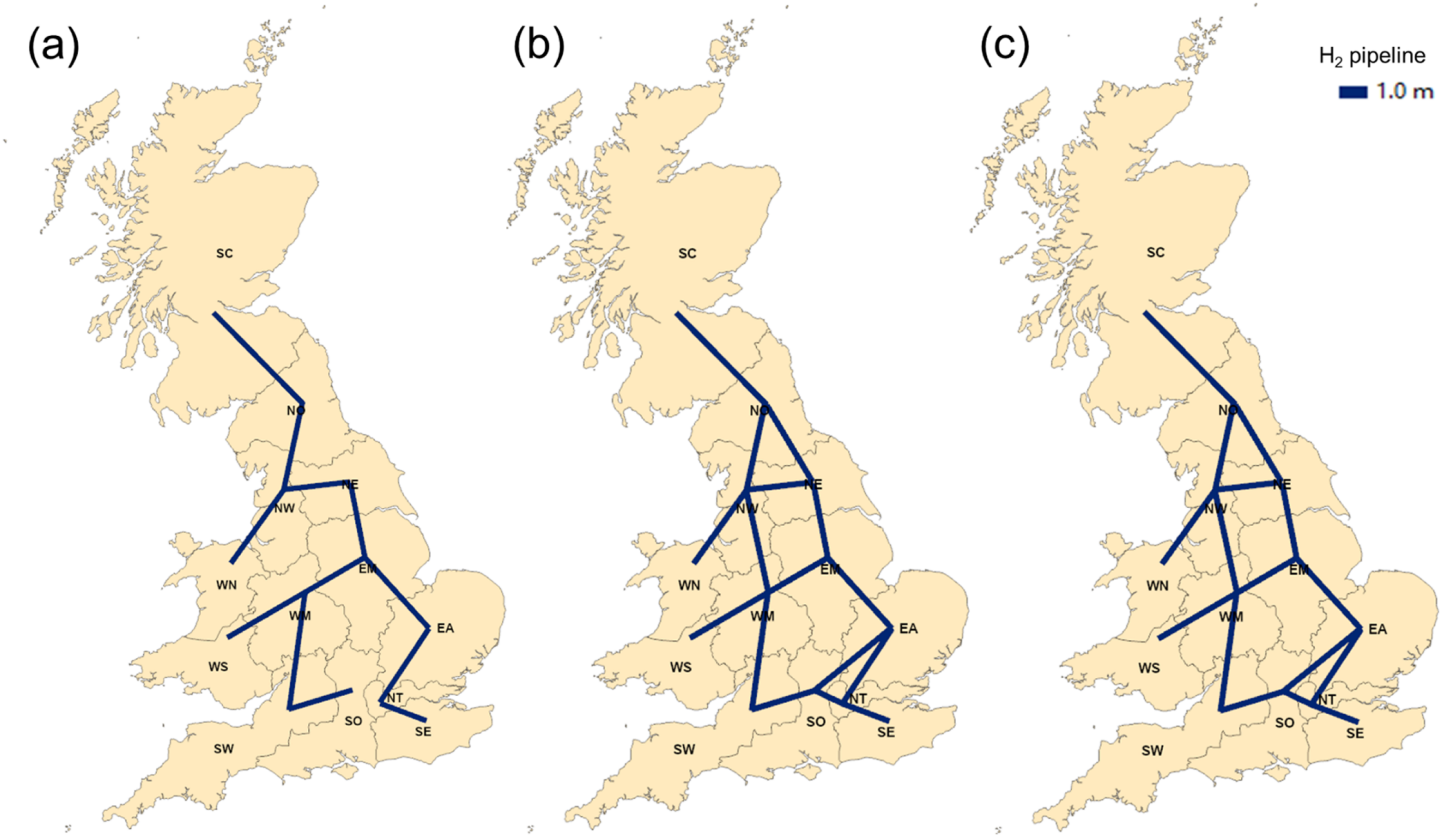
Hydrogen network maps (a) without any penetration constraint, (b) 10% penetration, and (c) 20% penetration.

Regarding hydrogen transmission, investments in water electrolysis can play an important role in decisions of the pipeline network. As illustrated in Figure 16, when there is no penetration of electrolysis, regions are not fully connected. However, water electrolysis production using electricity generated from renewable sources can have significant fluctuations, and thus, full connectivity of pipeline network is observed.

**Figure 17:**
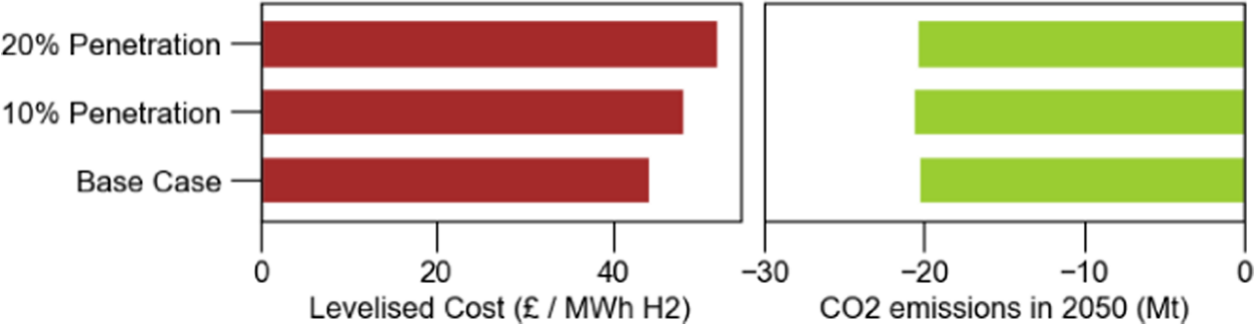
Levelized cost of hydrogen and CO_2_ emissions.

Figure 17 presents a comparison of levelized cost of hydrogen and total CO_2_ net emissions for the three cases. The levelized cost is increased with the rise of total electrolytic hydrogen production, as expected. On the other hand, CO_2_ emissions for all of the cases remain nearly the same across all scenarios across all cases. This is primarily because biomass gasification with CCS plays a crucial role in reducing emissions as it has a net-negative impact. Since all scenarios include the installation of 8 GW of gasification capacity, the consistent emissions across cases are reasonable.

MILP model size and computational performance of the three cases are summarized in Table 4. It should be noted that for both WE penetration cases, the total computational time exceeds 40h as the model is highly computational intensive.

**Table 4:** Model Size and Computational Performance

	base case		10% penetration		20% penetration	
case	step 1	step 2	step 1	step 2	step 1	step 2
continuous variables	854,104	854,104	854,104	854,104	854,104	854,104
discrete variables	246	384	246	384	246	384
equations	1,128,150	1,361,676	1,128,150	1,361,676	1,128,150	1,361,676
computational Time (h)	7.1	6.4	20.1	24.0	19.6	24.0
optimality gap (%)	1.34	3.38	1.11	5.95	3.52	6.47

**Figure 18:**
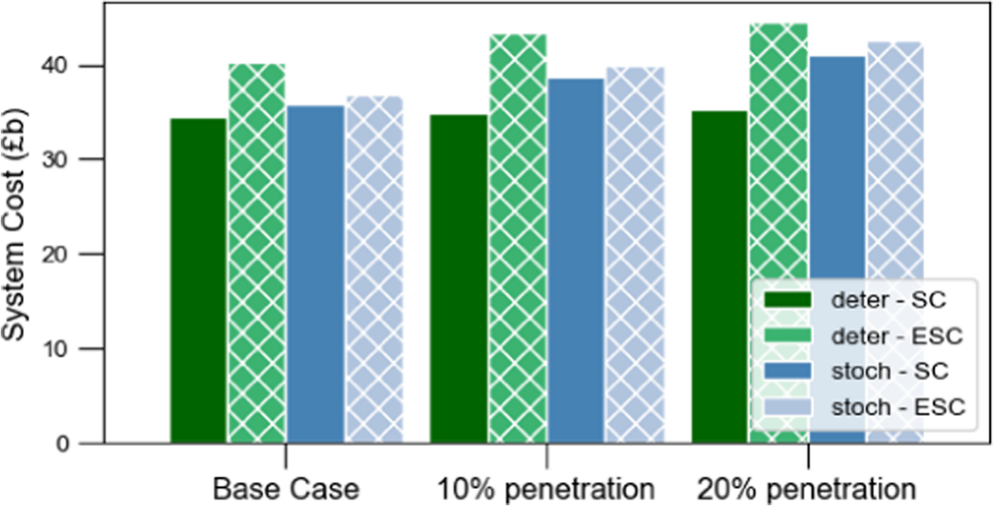
System cost and expected system cost for stochastic and deterministic approaches.

In Figure 18, system cost and expected system cost are illustrated for the three cases, comparing both deterministic and stochastic approaches. As WE penetration increases, the deterministic approach shows a marginal rise in system cost, while the expected system cost experiences a substantial increase. In contrast, as previously discussed, both system and expected cost in the stochastic approach grow proportionally with higher WE penetration. More specifically, in the 20% WE penetration scenario, the expected system cost increases by 20.5% for the deterministic approach, while it rises by only 3.4% for the stochastic approach.

The importance of the stochastic approach can be highlighted, particularly when WE is integrated in the production mix. Electrolysis is highly influenced by uncertain parameters such as technology costs and renewable sources availability. To this end, incorporation of uncertainty provides a more realistic strategy to investigate optimal pathways to achieve energy system decarbonisation.

## 6 Concluding Remarks

This work proposes a stochastic MILP framework to facilitate the investigation of optimal low-carbon hydrogen infrastructure design and operating decisions to meet hydrogen demand in GB. The spatially explicit model considers 3 time steps from 2030 to 2050 while typical days are selected for each calendar season of the time steps. An intraday time aggregation model is developed to define the optimal duration of time slices in each representative day. The continuity between the days within a time step is preserved to provide a more comprehensive and efficient storage strategy.

The two-stage stochastic model incorporates uncertainty in gas price, hydrogen demand, technology costs, and biomass and renewables availability. The combination of the aforementioned parameters results in a large scenario set. Forward scenario reduction is applied to decrease the number of scenarios, balancing the trade-off between the scenario set accuracy and computational time. The value of the stochastic approach is evident in its ability to achieve significantly lower expected costs compared to the corresponding deterministic approach, offering a risk-averse strategy.

The framework considers production, storage, and transmission of hydrogen as well as captured CO_2_ transmission and storage to obtain insights for policy making over the next decades. A total of 82 GW of production hydrogen capacity are commissioned for the base case consisting of ATR with CCS combined with BG with CCS offering the most cost-effective low-carbon infrastructure strategy, considering technology and feedstock cost and availability uncertainties. Additionally, storage capacity of 153 GWh is required to support the system mainly located in central England.

A sensitivity analysis is carried out to investigate how the penetration of electrolysis will affect the system. It is observed that there is an increase in production capacity, especially in north GB. Moreover, storage capacity is more equally located across GB, while more pipeline connections are essential to meet the demand with electrolytic hydrogen. Regarding system cost, a 20% penetration of electrolysis leads to a 17% increase in the levelized cost in comparison with the base case. Furthermore, the scenario-based approach is necessary to mitigate the risk, considering cost and availability uncertainties.

Future research will focus on the impact of economies of scale in infrastructure design decisions of a hydrogen system. In addition, the role of hydrogen in other sectors, such as the power sector, will be explored. In parallel, the investigation of new solution approaches and decomposition techniques will be conducted to deal with the combinatorial complexity and high computational times of the models.

## Appendix A

In this section, the proposed mathematical formulation is presented in detail based on the spatially explicit evolution model developed in our previous work,^45^ which has been modified as a two-stage stochastic multiperiod spatially explicit mixed-integer linear programming (MILP) model.

### Production Costs

A1

A2

### Storage Costs

A3

A4

### Transportation Costs

A5

A6

### Renewables Cost

A7

### Carbon Emissions Cost

A8

### Import Cost

A9

### Fuels Cost

The cost of the natural gas used in the reforming technologies can be calculated from eq A10.

A10

Similarly, the cost of biomass, which is used for biomass gasification, can be estimated from eq A11.

A11

### H_2_ Production

The hydrogen production rate is limited by an upper and lower bound according to eq A12.

A12

The operation of production plants is restricted by their ramp-up and ramp-down capabilities.

A13

A14

### H_2_ Storage

Moreover, upper bounds are imposed for the injection and removal rate.

A15

A16

where *Q*_*s*_^*I*max^ and *Q*_*s*_^*R*max^ are the maximum injection and removal rates for each storage type *s*.

### H_2_ and CO_2_ Pipeline

The maximum flow rate in the pipelines can be described by (eqs A17–A19) for the hydrogen flow rate (*Q*_gg′tchk_), onshore CO_2_ flow rate (*Q*_*gg*′*tchk*_), and offshore *CO*_2_ flow rate (*Q*_*grtchk*_).

A17

A18

A19

### CO_2_ Reservoirs

The reservoir CO_2_ inventory for each time step is equal to the inventory of the previous time step and the total flow rates to the reservoir.

A20

The inventory level is limited by an upper bound, as described in the constraint below.

A21

### H_2_ Imports

The import rate cannot exceed a percentage (ι) of the total hydrogen demand.

A22

### Electricity Production from Renewables

Hydrogen produced by water electrolysis is calculated according to eq A23.

A23

The electricity generation depends on the availability of the renewable sources *e* and the renewable capacity according to eq A24.

A24

The renewables capacity expansion is represented in eq A25, and it is limited by land availability, as described in eq A26.

A25

A26

### Fuel Consumption

Gas consumption depends on the production rate of reforming technologies, which include SMR and ATR and their efficiencies.

A27

Biomass consumption depends on the production rate of biomass gasification and the efficiency, and it is restricted according to biomass availability, as presented in eq A29.

A28

A29

### CO_2_ Emissions

The total CO_2_ emissions are calculated according to eq A30

A30

## Appendix B

The computational complexity of the proposed framework makes it imperative to reduce the model size. Historical demand and renewable sources availability have an hourly resolution. K-medoids clustering technique method is used for the selection of typical days per season, as described in Section 2.2. Moreover, further clustering to reduce the number of time slices within the day is necessary. To this end, a simple MILP model is formulated to merge the similar elements in the same intraday time slice.

In this case, hourly profiles of domestic, commercial, and industrial demand as well as solar, wind onshore, and offshore availability in all regions and clusters (seasons) are merged, decreasing the total intraday time slices from 24 to a smaller number. The aforementioned profiles for all of the regions are considered as different elements *w*.

The proposed model aims to minimize the summation Ω of distances *d*_*cwhl*_ of the new merged profiles from the original profiles for all of the clusters *c*, elements *w*, hours *l*, and time slices *h*, as presented in eq B1.

B1

A binary variable *Z*_*lh*_ is used to indicate in which time slice *h* the hour *l* is merged into. Thus, eq B2 guarantees that each hour *l* should be allocated to one time slice *h*. The continuity is enforced by eq B3 while the first hour *l* belongs to the first time slice *h*, as presented in eq B4.

B2

B3

B4

The distance of the normalized merged value *X_cwh_* of each cluster *c*, element *w*, and time slice *h* and initial normalized profiles PN_*clw*_ of each cluster *c*, hour *l*, and element *w* are calculated, as presented in eqs B5 and (B6).

B5

B6

The mathematical framework is formulated as an MILP model minimizing the distances subject to eqs B1–(B6). The model is implemented in GAMS version 46.1.0^60^ and solved with Gurobi version 11.0.0 using 0% relative optimality gap as the termination criterion.

## Appendix C

The relative probability distance (RPD) between the reduced and original scenario sets is a metric to evaluate the quality of reduced scenario set in comparison with the original one. It can be defined as the absolute probability distance (RD) divided by the absolute probability distance when reduced scenario consists of one scenario (RD^1^), as presented in eq C1.

C1

Probability distance between initial scenario set *S*^init^ and reduced set *S*^*N*^ of scenarios *N* can be calculated from the formula, as presented in eq C2.

C2

where π_*s*_^init^ stands for the original probability of scenario *s* and γ_pss′_ stands for the distance of random variable *p* normalized realizations between scenario *s* and *s*′.^50,62^

Marginal relative probability distance (mRPD) is defined as the difference between RPD^*N*^ when scenario reduction selects *N* scenarios and RPD^*N*–1^ when scenario reduction selects *N*–1 scenarios.^50^ More specifically, it represents the marginal RPD reduction of the original and reduced scenario set when *N* scenarios are selected, as described in eq C3.

C3

## Appendix D

In this Appendix, hydrogen demand, biomass availability, gas price, and technology costs data are presented in Tables D1, DD2, DD3, DD4 and DD5.

**Table D1:** Demand Data Across Scenarios in 2050 [TWh]^40,55^

	scenario				
sector	1	2	3	4	5
residential	145	43	72	144	216
commercial	46	21	24	47	71
transportation	138	86	112	112	112
industrial	88	54	22	45	67
total	417	204	230	348	466

**Table D2:** Biomass Availability [TWh]^56^

	scenario		
year	low	base	high
2030	45	74	103
2040	40	67	93
2050	49	83	117

**Table D3:** Gas Price [£/MWh]

	scenario				
year	1	2	3	4	5
2030	11.7	22.2	32.8	43.3	53.8
2040	12.6	23.8	34.8	45.8	56.8
2050	13.4	25.5	37.5	49.6	61.7

**Table D4:** Cost of Water Electrolysis Plant in Time Step *t*(58)

		2030	2040	2050
capital cost [£k/MW]	low	494	442	429
	base	671	633	613
	high	975	910	878
operating fixed cost [£k/MW/y]	low	29.9	28.7	28.7
	base	30.1	29.6	29.3
	high	34.1	32.9	32.3
operating variable cost [£/MWh]	low	2.9	2.8	2.8
	base	4.2	4.0	4.0
	high	6.1	5.8	5.7

**Table D5:** Capital Cost of Renewable Farm Type *e* in Time Step *t* [£k/MW]^58^

		2030	2040	2050
solar	low	375	275	275
	base	420	320	320
	high	485	385	385
wind onshore	low	993	993	993
	base	1304	1304	1304
	high	1502	1502	1502
wind offshore	low	1316	1216	1216
	base	1874	1824	1824
	high	2204	2534	2534
